# Supplementary material for: Patterns of Intron Gain and Loss in Fungi
Source: PLoS Biol. 2004 Nov 30;2(12):e422. doi: 10.1371/journal.pbio.0020422 (PMC532390; doi:10.1371/journal.pbio.0020422)
Supplement: Table S1 — Also available at http://genes.mit.edu/NielsenEtAl/. (4.3 MB ZIP). [file pbio.0020422.st001.zip › NielsenEtAl/html/1370.html]

AN2847.1.NCU06664.1.MG05995.1.FG10344.1


```
 CLUSTAL W (1.82) Multiple Sequence Alignments - Introns Inserted


Sequence 1: NCU06664.1	239 aa
Sequence 2: MG05995.1	243 aa
Sequence 3: FG10344.1	249 aa
Sequence 4: AN2847.1	255 aa
Alignment Length: 271 aa
Number Identitical Residues: 79 aa
Alignment Score (without introns) 4609


MG05995.1 	--MAQ-MIPQAVLDQLKAQIDQDSKTKE~ALGDITEKLEREV~AYSQGVISRVHATRVAD
NCU06664.1	--MAPPMIDPALFDQLKDNIEKDSSIRK~DLEQIIEELNQNV~SYTQGVLTKIHSTPRSK
FG10344.1 	MSSETPLLDPSLFSHLQEKLDEETSVRD~NLTQIIQRLERAV~ATAQGLLSRVHSTPRAR
AN2847.1  	------MIDRVIFENLQAKIDEEAAVRD0ELRDIVQNLSRKG1RSTQAVLSRAHSTPEAQ
          	      ::   ::.:*: ::::::  :.  * :* :.*.:     :*.:::: *:*  : 

MG05995.1 	Y~AAALLPQLEAAIKDMIATTKALEEEASKHPYYK2YNFKWTRHVRGA~IFTIVLCAFLG
NCU06664.1	Y1P-ALLSQVESGIKKELETTTRLAQFASQYPYYK2YNYKWGRTLQDA~IATVLLHAWLG
FG10344.1 	Y1P-ALVSQVEDAIKEEVTIVKELSEVASQHPYYK2YNQKWARTVQNA~IGTAVYTAWLG
AN2847.1  	L1Q-PVLDDATKEILAQKEEITRLKAVADRHPFYK~YNGVWSRDLQNL0VASIELCAWLG
          	    .:: :    *       . *   *.::*:** **  * * ::.  : :    *:**

MG05995.1 	GLGNETKPGELGRLLSLEEVGAVLQ---------------------V1PVNIQDRDVFHI
NCU06664.1	GMGSDSKPGEVGRLLTLEEVGHVFE---------------------V1PVNLKDRDAFHI
FG10344.1 	GLGSDSQPASLGRLLTLEQVGAVFQ---------------------V1PTNLKDRDAFHF
AN2847.1  	GL-QEFKGSESASFLTMEEVGKFLESMALLCHFDCVSAWYLTRIRAV~PVNLKEEDAFHL
          	*: .: : .. . :*::*:** .::: :  .  .. ::   :   :* *.*:::.*.**:

MG05995.1 	TIEEYLLSLTDLTNEL~SRLTTNTVTLGDFEMAVRISSFVRDLHAGFQLLNLKNDILRKR
NCU06664.1	TIEEYLLALISVVEDL~SRLAMNSVTLGDTELAVQISGFIKDLHGGFQMLNLKNDILRKR
FG10344.1 	TIEEYLLSLTDLTNEL~ARLAPNAVTHGDFELPLVISAFIKDLFAGFQLLNLKNDILRKR
AN2847.1  	TLEEYLLALISMIEEL0ARLAVNAVTLGDYGRPTVIGNFIKELFNGFQLLNLKNDVLRKR
          	*:*****:* .: ::* :**: *:** **   .  *. *:::*. ***:******:****

MG05995.1 	VDSVKYD~VKKVEDVVYDLSLRNLIP---ARQSAGAAQ-
NCU06664.1	VDSVKYA~VKKVEDVVYDLSLRNLIP---AKDAQ-----
FG10344.1 	ADAVKYD~VKRVEDVVYDLSLRGLVKRPGEGDTEMAAAE
AN2847.1  	SDAIKYS0VKKVEDVVYDLSLRNLIP---KGQGV-----
          	 *::**  **:***********.*:      :
```
